# mTOR Inhibitors in Tuberous Sclerosis Complex

**DOI:** 10.2174/157015912804143595

**Published:** 2012-12

**Authors:** Paolo Curatolo, Romina Moavero

**Affiliations:** Pediatric Neurology Unit, Neuroscience Department, Tor Vergata University Hospital, Rome, Italy

**Keywords:** Angiomyolipoma, tuberous sclerosis complex, everolimus, mTOR inhibition, subependymal giant cell astrocytoma, epilepsy.

## Abstract

Tuberous sclerosis complex (TSC) is a genetic multiple organ system disorder that is characterized by the development of tumor-like lesions (hamartomas) and neurodevelopmental disorders. Mutations in the *TSC1 *and *TSC2 *tumor suppressor genes occur in the majority of patients with TSC, resulting in hyperactivation of the mammalian target of rapamycin (mTOR) signaling pathway and subsequent abnormalities in numerous cell processes. As a result, mTOR inhibitors such as sirolimus and everolimus have the potential to provide targeted therapy for patients with TSC. Everolimus is the first mTOR inhibitor approved as a treatment option in the USA and in Europe for patients with subependymal giant-cell astrocytomas (SEGAs) associated with TSC. The clinical evidence to date supports the use of mTOR inhibitors in a variety of TSC-associated disease manifestations, including SEGAs, renal angiomyolipoma, skin manifestations, and epilepsy. Furthermore, ongoing clinical trials evaluating mTOR inhibitors in TSC are underway, and the results of these studies are expected to provide further evidence that will firmly establish their role in this setting. This article will discuss the role of the mTOR pathway in TSC and review the pharmacokinetics, pharmacodynamics, clinical efficacy, and tolerability of mTOR inhibitors, along with their current place in clinical practice.

## INTRODUCTION

Tuberous sclerosis complex (TSC) is a variably expressed autosomal dominant genetic disorder that is pathologically characterized by the presence of benign, non-invasive, tumor-like lesions (hamartomas) in multiple organ systems, including the brain, heart, skin, kidney, lung, and liver [1]. This relatively common single gene disorder affects both adults and children, with an estimated birth incidence of approximately 1 in 6000 [2, 3]. Population-based studies have reported the estimated prevalence of TSC to be between 1:14,000 and 1:25,000 [4, 5]. Evidence suggests that patients are diagnosed most frequently at <15 months old; prevalence of TSC decreases as age increases, with prevalence being 1:14,000, 1:19,000 and 1:24,000 for those aged <6, 12 and 18 years old, respectively [4, 5].

In TSC, mutations in one of the two tumor suppressor genes, *TSC1* (encoding hamartin) or *TSC2* (encoding tuberin), are found in more than 85% of cases [6]. Hamartin and tuberin are involved in the regulation of cell proliferation and differentiation, forming a physical and functional complex that activates GTPase, keeping the protein Ras homolog enriched in brain protein (RHEB) inactive in order to inhibit the mammalian target of rapamycin (mTOR) pathway. The mTOR pathway is responsible for protein and lipid biosynthesis and growth factor-related cell cycle progression. Under normal circumstances, hamartin and tuberin are activated in unfavorable conditions to prevent substrate use *via *biosynthetic processes mediated by the mTORC1 complex, which includes mTOR, raptor (mTOR regulatory-associated protein of mTOR), mLST8, and PRAS40 (proline-rich Akt substrate 40) [1, 7]. Therefore, *TSC1* or *TSC2* mutations in patients with TSC gives rise to hyperactivation of the mTOR pathway, resulting in a downstream kinase signaling cascade that can consequently lead to abnormalities in numerous cell processes, including cell cycle progression, transcription, translation, and metabolic control [1, 7, 8]. 

One of the most commonly affected organ systems in TSC is the central nervous system (85-90% of children and adolescents), [1] which can cause disabling neurological manifestations, including epilepsy (66-93% of patients with TSC), subependymal nodules (SENs; 90-100%), subependymal giant cell astrocytomas (SEGAs; 5-20%), mental retardation (44-64%), and infantile spasm (45%) [9]. SEGAs are slow-growing glioneuronal tumors located adjacent to the foramen of Monro, and their continued growth can block cerebrospinal fluid circulation, leading to an increase in intracranial pressure [8, 10]. 

Currently, it is not possible to identify asymptomatic SEGAs that are likely to cause problems later in life [1]. Therefore, magnetic resonance imaging (MRI) of the brain should be conducted in patients with a definite diagnosis of tuberous sclerosis and risk factors for developing astrocytomas [1, 10, 11]. The surgical resection of intracranial lesions is the current standard treatment for patients with symptomatic SEGA in TSC [1, 12]. Given that the underlying abnormality in TSC is mTOR hyperactivity, the possibility of the mTOR pathway as a therapeutic strategy has been investigated as an alternative nonsurgical treatment of SEGA in patients with TSC [8]. 

mTOR inhibitors sirolimus (rapamycin; Rapamune^®^) and everolimus (RAD001; Afinitor^®^ [USA]; Votubia^®^ [EU]) have been investigated in patients with TSC, most extensively as an alternative nonsurgical intervention for TSC-related SEGA. Currently, everolimus is the only mTOR inhibitor approved for the treatment of TSC. It has been approved in various countries for the treatment of patients aged ≥3 years with TSC-related SEGA who require therapeutic intervention, but are not candidates for curative surgical resection [13, 14].

This review will focus on the role of mTOR inhibitors in the treatment of tuberous sclerosis. We will discuss the role of the mTOR pathway in TSC, the pharmacology of mTOR inhibitors, preclinical and clinical trials investigating their role in TSC, and address their use, efficacy, safety, and place in clinical practice.

## PHARMACOLOGICAL ASPECTS OF MTOR INHIBITORS

### Pharmacodynamic Properties

Sirolimus is a macrolide antibiotic produced as a fermentation product of *Streptomyces hygroscopicus *[15]. The drug was first developed as an antifungal agent, but was later found to have potent antiproliferative and immunosuppressive properties [16]. Everolimus is an orally bioavailable, structurally similar derivative of sirolimus that also exhibits antiproliferative and immunosuppressive effects [17]. Everolimus was developed in an attempt to improve upon the pharmacokinetics of the sirolimus, and has been shown to provide greater stability, solubility, and more favorable pharmacokinetics [17]. Both sirolimus and everolimus have the potential to treat the underlying cause of TSC by inhibiting the mTOR pathway, and the molecular structures of these two agents are shown in Fig. (**1**).

**Fig. (2):**
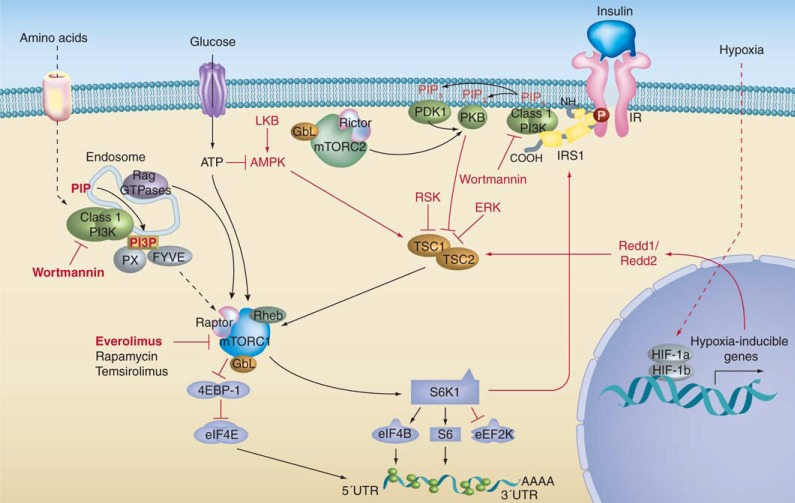
Summary of the role of the mammalian target of rapamycin (mTOR) signaling pathway in the development of tuberous sclerosis complex (TSC) and inhibition by mTOR inhibitors. Reproduced from Expert Review of Anticancer Therapy, August 2011, Vol. 11, No. 8, Pages 1181-1192 with permission of Expert Reviews Ltd [19].

The mechanisms of action for sirolimus and other rapalogs (i.e. everolimus, temsirolimus, and ridaforolimus) are similar (Fig. **2**). Each drug forms a complex by binding to the intracellular binding protein FK506-binding protein (FKBP12), which subsequently binds to mTOR at the FKBP12-rapamycin binding domain, thus inhibiting downstream signaling events [18]. Both sirolimus and everolimus exert their inhibitory effects on mTOR-regulated processes by reducing the phosphorylation of downstream mTOR effectors, including the translational repressor eukaryotic elongation factor 4E binding protein 1 (4EBP1) and the S6 ribosomal protein kinase 1 (S6K1) [8, 19, 20]. This inhibitory effect disrupts S6K1 and 4EBP1 function, which are responsible for the translation of mRNA encoding pivotal proteins that are involved in cell cycle regulation, glycolytic activity, angiogenesis, cell size control, and cellular growth [8, 19, 20]. 

Another important mechanism in which sirolimus and everolimus exert their antitumor effects is their ability to reduce the expression of factors involved in the angiogenic process, such as vascular endothelial growth factor (VEGF). VEGF promotes neovascularization, enables growth, and it has been thought to play a significant role in the development of solid tumors [20]. Furthermore, an upregulation of VEGF production has been shown to be responsible for extensive abnormal vascularization of TSC-related tumors, thereby increasing the growth of these tumors [21]. Sirolimus and everolimus have been shown to reduce the expression of hypoxia-inducible factor-1 (HIF-1; and consequently VEGF), and inhibit endothelial cell, smooth muscle cell, and pericyte formation *in vitro *[20, 22-25].

### Pharmacokinetic Properties

The main pharmacokinetic properties of sirolimus and everolimus are summarized in Table **1** [19].

Sirolimus is available orally as a solution or in tablet form, and bioequivalence has been demonstrated for the two formulations in a study of stable renal allograft recipients [26]. Although the oral bioavailability of sirolimus is low (approximately 15%), it is rapidly absorbed with an estimated t_max_ of 2 hours [27]. The wide interpatient variability of sirolimus is largely attributed to the effects of intestinal cytochrome p450 3A enzymes (CYP3A) and P-glycoprotein activity on sirolimus absorption [16]. The coadministration of sirolimus and cyclosporine in renal transplant patients alters the bioavailability of sirolimus, increasing both the C_max_ and area under the concentration-time curve (AUC) of sirolimus [28]. Sirolimus absorption was also shown to be reduced by the intake of a high-fat meal, although a 35% increase in AUC was reported [29]. A large volume of distribution (approximately 12 L/kg) is seen with sirolimus, the majority of which is distributed into red blood cells (approximately 95%) [18, 30]. Sirolimus is mainly metabolized *via *hepatic CYP3A enzymes, is primarily excreted *via *the fecal route, has a clearance that ranges from 1.45 to 6.93 mL/min/kg), and a half-life of approximately 62 hours [18, 27]. 

Everolimus, a derivative of sirolimus, shares many characteristics with the later agent, including extensive distribution of the drug into red blood cells, [31] metabolism by hepatic CYP3A enzymes, [13, 14, 17] and primarily fecal excretion [17]. However, everolimus was developed to improve upon the pharmacokinetics of sirolimus and is associated with several favorable pharmacokinetic parameters over the latter agent, including a favourable blood-brain partition coefficient, [32] greater bioavailability, [18, 33] and greater water solubility [34, 35]. In rats, the bioavailability of everolimus was greater, compared with sirolimus (16% vs. 10%) [36]. Everolimus was also associated with a shorter half-life than sirolimus, suggesting that a steady state concentration is achieved more rapidly with everolimus [33]. In healthy volunteers and patients with solid tumors, everolimus was rapidly absorbed, with a t_max_ that ranged from 30 min to 1 hour and a half-life of approximately 30 hours [37-39]. The C_max_ of everolimus was reduced by 54% and the overall AUC by 22% when a high-fat meal was consumed by healthy volunteers [13]. In contrast, an estimated 2-fold increase in the AUC value was reported in patients with moderate hepatic impairment, compared with healthy volunteers [13, 14, 40].

## PRECLINICAL STUDIES IN MODELS OF TSC

Sirolimus and everolimus have both undergone extensive *in vitro *and *in vivo* investigation in models of TSC.

A number of studies have investigated the effect of sirolimus on controlling the appearance and progression of TSC-related tumors. The inhibitory effects of sirolimus on mTOR-dependent signaling have been demonstrated *in vitro *[25, 41]. Moreover, an *in vitro *study in *TSC1*- and *TSC2-* null mouse embryo fibroblasts observed that TSC gene products regulate VEGF production *via *the mTOR signaling pathway and demonstrated that combination treatment with interferon-γ and sirolimus was associated with a reduction in VEGF levels and induction of apoptosis in these cells [21]. The same study also evaluated short-term sirolimus treatment in *TSC1 *knockout mice, which resulted in evidence of changes in tumor morphology and a reduction in serum VEGF levels [21]. These results suggest that mTOR inhibition with sirolimus may be lead to direct tumor cell killing and the inhibition of TSC lesion development through impairment of VEGF production.

In another animal model, primary tumors from the Eker rat model of TSC demonstrated elevated expression of phosphorylated mTOR and its effectors (S6K1, S6 ribosomal protein, and eukaryotic translation initiation factor 4 gamma) [42]. In this study, the short-term treatment of Eker rats with sirolimus was associated with a reduction in cell proliferation, induction of apoptosis, and significant tumor response [42]. A second study assessed the effects of sirolimus administration on Eker rats with pituitary and renal tumors [43]. Treatment with sirolimus was associated with clinical improvement, a marked decrease in tumor volume, and prolonged survival [43]. These improvements in tumor response were also accompanied by down-regulation of ribosomal S6K activity, cell size reduction, and induction of apoptosis [43].

Everolimus has demonstrated the ability to inhibit cell growth and proliferation in a number of *in vitro *studies in various human tumor cell lines, including breast cancer, [23, 44-46] nasopharyngeal carcinoma, [47] ovarian cancer, [24, 46] and mantel cell leukemia [48]. Furthermore, a study of human umbilical vein endothelial cells showed that everolimus effectively inhibited mTOR signaling and increased the apoptosis of endothelial cells in response to radiation [25].

The success of these *in vitro *studies was accompanied by several animal studies that demonstrated the favorable effects of everolimus in models of TSC. Studies conducted in a murine neuronal models of TSC reported that everolimus was associated with complete and sustained inhibition of mTOR dependent-signaling pathways, resulting in reductions in cell size, neurofilament abnormalities, and improvements in myelination [49, 50]. As a result, improvements in both phenotype and survival were seen in these animals. Everolimus also demonstrated antiangiogenic activity in a study of a murine glioma tumor model, which reported a reduction in tumor vascularization [25].

Studies involving mouse models have demonstrated the ability of mTOR inhibitor therapy to improve learning deficits and reduce seizures [51]. The poor spatial learning observed in adult *TSC2^+/-^* mice was reversed following a brief treatment with sirolimus [52]. The suppression of seizures *via *mTOR inhibition with sirolimus has also been demonstrated in a mouse model of cortical dysplasia [53]. Furthermore, a mouse model of TSC showed that the early treatment of these animals with sirolimus prevented the development of epilepsy; [54] late treatment with sirolimus in mice who had already developed epilepsy also provided seizure suppression. 

The use of both *in vitro *and rodent models of TSC has been major tools in the assessment of potential therapies for TSC. Sirolimus and everolimus have both demonstrated therapeutic benefit in studies involving these models of TSC, which has been crucial in the development of the current set of clinical trials with these agents in patients with TSC.

## CLINICAL STUDIES OF mTOR INHIBITION IN TSC

### Sirolimus

Two nonrandomized, open-label, prospective studies investigated the efficacy and tolerability of sirolimus in TSC patients with renal AMLs and lymphangioleiomyomatosis (LAM). A phase I/II proof-of-concept study was conducted in 25 patients with TSC and LAM who received an initial sirolimus daily dose of 0.25 mg/m^2^, followed by up-titration at 2 weeks, 2 months, and 4 months to plasma sirolimus levels of 1-5, 5-10, and 10-15 ng/mL, respectively [55]. Renal AML volume was significantly decreased to approximately 53% of the baseline value (p<0.001) after 12 months of treatment, but tumor regrowth to 86% of the baseline value (p=0.005) was observed at 24 months [55]. Significant improvements in pulmonary function were also seen in some patients. 

The phase II, multicenter Trial of Efficacy and Safety of Sirolimus for Treatment of Angiomyolipoma in Tuberous Sclerosis and Sporadic LAM (TESSTAL) assessed a total of 16 patients with TSC or sporadic LAM and renal AMLs [56, 57]. Patients received sirolimus for up to 2 years, with an initial oral daily dose of 0.5 mg/m2, titrated to a trough blood level of 3-6 ng/mL and 6-10 ng/mL. An interim analysis at 12 months reported a reduction in tumor volume of >50%, but the tumor size increased upon treatment cessation [57]. The overall response rate (as measured by Response Evaluation Criteria in Solid Tumors [RECIST]) was 50% and a response rate of 80% was reported in the per-protocol group; at 24 months, a partial response was observed in 40% of patients who remained in the trial [56]. No significant improvement in pulmonary function was seen in this study, but recall memory was improved in 7 out of 8 patients with TSC [56].

Several other clinical stidues have demonstrated the efficacy of sirolimus in reducing the size of a range of different tumor types in patients with LAM [12, 58-62].

The efficacy of sirolimus has also been observed in other TSC-related lesions, including pulmonary fibrosis, skin manifestations, and facial angiofibromas. Sirolimus was associated with significant responses to cases involving chest wall fibromatosis and skin lesions [63, 64]. A number of case reports have also demonstrated significant reductions in facial angiofibromas following treatment with sirolimus in patients with TSC [65-68].

### Everolimus

There is growing evidence from clinical studies that everolimus may be considered as a therapeutic option and an alternative to surgery in selected patients with TSC-associated lesions such as SEGAs. 

### Phase II Trial C2485

C2485 was a pivotal phase I/II, prospective, single-center, open-label trial assessed the efficacy and safety of oral everolimus in 28 patients (median age 11 years; range 3-34 years) with TSC-related SEGAs [12]. Treatment was initiated at a daily dose of 3 mg/m^2^, which was titrated to maintain a serum trough concentration of 5-15 ng/mL. At the completion of the core 6-month treatment phase, [12] patients were eligible to transition into an open-label extension phase [69]. The median treatment duration of everolimus was 21.5 months (range 4.7-34.4 months).

At 6 months, everolimus was associated with a statistically significant and clinically relevant reduction in the volume of the primary SEGA lesion (primary endpoint; -0.80 cm^3^; p<0.001); [12] the mean reduction in SEGA volume assessed by independent central review was 1.15±1.42 cm^3^ (p<0.001) [12]. Mean reductions of ≥30% and ≥50% from baseline in the volume of the primary SEGA lesion were observed in 21 (75%) and 9 (32%) patients, respectively [12]. The overall frequency of clinical and subclinical seizures was also significantly decreased at 6 months (median change -1 seizure; p=0.02) [12]. Furthermore, the mean number of electrographic seizures, as assessed by 24-hour video electroencephalography (EEG; available for 16 patients), decreased from 6.30 seizures/24 hours at baseline to 2.75 seizures/24 hours at 6 months (p=0.022) [69]. No patients developed new lesions, worsening hydrocephalus, or evidence of increased intracranial pressure, and no patients required surgical resection or other SEGA treatment [12]. Interestingly, the lesions of patients with facial angiofibromas had almost disappeared after 6 months of treatment in 13 of these 15 patients [12]. Quality-of-life following everolimus therapy was assessed using the validated Quality-of-Life in Childhood epilepsy questionnaire, which showed an improvement from baseline at 3 and 6 months [12].

Of the 28 patients enrolled into the core treatment phase, 27 transitioned into the long-term, open-label extension phase of this pivotal study [69]. Most importantly, the reduction in SEGA volume was maintained in patients who received everolimus for ≥3 years, and a reduction of ≥30% in the volume of the primary SEGA lesion was maintained for a median duration of 23.8 months [69]. After 24, 30, and 36 months of treatment with everolimus, a reduction from baseline of ≥50% in the volume of the primary SEGA lesion occurred in 50%, 41%, and 56% of patients, respectively [69]. Furthermore, diffusion tensor imaging showed improvements from baseline in the integrity of normal-looking white matter at 12 and 18 months [70].

### Phase III EXIST-1 Trial in Patients with SEGA

EXIST-1 (Examining everolimus In a Study of TSC) was a phase III international, multicenter, double-blind, randomized, placebo-controlled trial that evaluated the efficacy and safety of everolimus in 117 patients (median age 9.5 years; range 0.8-26.6 years) with SEGA associated with TSC [69]. In this study, eligible patients were randomly assigned to placebo (n=39; median treatment duration 36.1 weeks) or everolimus (n=78; median 41.9 weeks), which was initiated at a daily dosage of 4.5 mg/m^2^ and titrated to target trough levels of 5-15 ng/mL until SEGA progression or unacceptable toxicity. Placebo recipients with SEGA progression were unblended and offered open-label everolimus therapy. The primary endpoint of EXIST-1 was the proportion of patients with a SEGA response (confirmed by MRI 8-12 weeks after the response), defined as a reduction from baseline of ≥50% in the sum volumes of all target SEGA lesions, non-worsening of non-target SEGA lesions, no new SEGA lesions ≥1 cm, and no new/worsening hydrocephalus. Key secondary endpoints included the change from baseline in seizure frequency at 6 months (assessed by video EEG), time-to-SEGA progression, and the skin lesion response rate (assessed by the Physician’s Global Assessment of Clinical Condition every 3 months). 

Everolimus was associated with a significantly greater overall SEGA response rate, compared with placebo (35% vs. 0%; p<0.0001); this benefit was consistent across all patient subgroups analyzed [69]. No differences in the frequency of seizures were seen between the two treatment groups at 6 months. The median time-to-SEGA progression was not reached, but the estimated progression-free rate at 6 months was significantly higher with everolimus (100% vs. 86%; p=0.0002). Everolimus was also associated with greater partial skin lesion response (42% vs. 11%; p=0.0002) and AML response (53% vs. 0%) rates, compared with placebo. 

### Phase III EXIST-2 Trial in Patients with AML

The phase III EXIST-2 trial is an ongoing, international, multicenter, double-blind, randomized, placebo-controlled study that assessed the efficacy and safety of everolimus in 118 patients (median age 31 years; range 18-61 years) with AML associated with TSC or sporadic LAM (LAM). Patients were randomly assigned to receive placebo (n=39) or everolimus 10 mg/day (n=79) once daily for a median treatment duration of 34.0 and 38.1 weeks, respectively. The primary efficacy endpoint for EXIST-2 was the proportion of patients who achieved a best overall AML response (confirmed by kidney computed tomography/MRI 8-12 weeks after the response), which was defined as a reduction from baseline of ≥50% in the sum of volumes of all target AML lesions, no new lesions ≥1 cm in the longest diameter, no kidney volume increase of >20% from nadir, and no AML-related bleeding grade ≥2 (defined by the National Cancer Institute Common Terminology Criteria for Adverse Events, version 3.0). Key secondary endpoints included the time-to-AML progression, skin lesion response rate, and response defined as complete (absence of disease) or partial (≥50% improvement) clinical response. 

Everolimus was associated with a significantly greater AML response rate, compared with placebo (41.8% vs. 0%; p<0.0001); this benefit was consistent across all patient subgroups analyzed. The median time-to-AML progression was 11.4 months in the placebo group, but was not reached in the everolimus treatment group (hazard ratio 0.08; 95% CI 0.02-0.37; p<0.0001); estimated 6-month progression-free rates of 98.4% and 83.4% were reported in the everolimus and placebo groups, respectively. Furthermore, partial skin lesion response occurred in 26% of everolimus recipients, compared with 0% of placebo recipients (p=0.0002).

### Ongoing Studies

Several ongoing clinical studies are currently underway to investigate the use of mTOR inhibitors such as sirolimus and everolimus in a range of TSC-related manifestations (Table **2**).

The potential for mTOR inhibitors such as sirolimus and everolimus to be of therapeutic benefit in non-SEGA manifestations that are associated with TSC is being investigated in a number of ongoing clinical trials. An open-label, phase IV trial assessing the efficacy and safety of sirolimus in 18 patients with TSC-related AMLs was recently completed (ClinicalTrials.gov Identifier: NCT01217125) and will report the changes in AML volume, skin lesion response, and the incidence of AML complications of a period of 2 years. Preliminary results from 17 patients enrolled in this study show that 1 year of sirolimus treatment reduced AML volume by 50% in 82.4% of tumors; AML volume decreased an average of 66.3% in 12 months [71]. Skin lesions also improved with sirolimus treatment. The most frequent adverse events included oral aphthous ulcers (5/17), microcytosis and hypochromia (3/17), and hypertriglyceridemia (3/17) [71].

Ongoing studies are also being conducted to investigate everolimus in non-SEGA indications associated with TSC. An open-label, single-arm, phase I/II trial has been initiated to assess the efficacy of everolimus in patients with AML associated with TSC or sporadic LAM (ClinicalTrials.gov Identifier: NCT00457964). An extension-phase for this study has also been initiated to assess the long-term safety and efficacy of everolimus in these patients (ClinicalTrials.gov Identifier: NCT00792766).

The efficacy and safety of everolimus in AML associated with TSC or sporadic LAM is also being evaluated in the phase III EXIST-2 trial, which has a planned study completion date of January 2014 (ClinicalTrials.gov Identifier: NCT00790400); positive preliminary results have been reported to date. Finally, an open-label, phase I/II study has been initiated to determine if everolimus can reduce the number of epileptic seizures in patients with TSC (ClinicalTrials.gov Identifier: NCT01070316). The results of these studies will hopefully provide data that will demonstrate the effectiveness of mTOR inhibitors in a broad spectrum of TSC manifestations.

Everolimus is also being investigated for the treatment of neuroendocrine tumors (NET), which exhibit constitutive activation of the mTOR signaling pathway. Indeed, everolimus (Afinitor^®^; Novartis Pharmaceuticals) was recently approved by the US FDA in 2011 for the treatment of progressive NET of a pancreatic origin [72]. This approval was based on phase III data from the ongoing RADIANT-3 (RAD001 In Advanced Neuroendocrine Tumors) study, [73] which reported that everolimus significantly doubled the time without tumor growth (median 4.6 to 11.0 months) and was associated with a 65% reduction in the risk of cancer progression (ClinicalTrials.gov Identifier: NCT00510068). 

## SAFETY AND TOLERABILITY

In general, mTOR inhibitors are well tolerated and have similar AE profiles [18]. The majority of AEs associated with the mTOR inhibitors are linked to the immunosuppressive action of this drug class, and include aphthous ulcers, fatigue, rash, mucositis, anorexia, gastrointestinal effects such as diarrhea and nausea, arthralgias, thrombocytopenia, and effects on lipid metabolism [74]. In most cases, these AEs are self-limiting and can be managed by dose reductions or cessation [18]. Potentially serious AEs may include upper respiratory tract infections as well as non-infective pneumonitis [75, 76] and dramatic elevations in serum cholesterol and lipoprotein levels, which may require dietary adjustment or the use of cholesterol-lowering medication [12, 74].

Two open-label, prospective studies of sirolimus in TSC patients with renal AMLs and LAM reported relatively high rates of AEs [55-57]. In the phase I/II proof-of-concept study, patients with TSC and sporadic LAM who were treated with sirolimus reported a high rate of AEs, which included infections (31 events in 17 patients), aphtous ulcers (22 events in 17 patients), and diarrhea (12 events in 7 patients) [55]. A total of 6 serious AEs that were probably or possibly related to sirolimus occurred in 5 patients during this study. In the second study, which was a phase II trial in patients with AML associated with TSC or sporadic LAM, [56, 57] the majority of AEs were mild and were consistent with the AE profile of sirolimus. In this study, the most frequently reported AEs included oral mucositis (6 patients), respiratory infections (5 patients), and proteinuria (5 patients); 3 out of 7 serious AEs were considered to be sirolimus-related [56, 57].

Clinical trials of everolimus in patients with TSC-associated SEGA have shown that this agent is generally well tolerated. In the core phase of the open-label phase II study (C2485) of everolimus in TSC patients with SEGA, the most commonly occurring AEs included self-limiting events such as stomatitis (22 events), upper respiratory infection (22), sinusitis (11), otitis media (10), and pyrexia (10) [12]. A total of 4 patients had serious AEs, which included recurrent upper respiratory infection (viral bronchitis), pneumonia, vomiting, and convulsions [12]. AEs reported in the core study were consistent with the known AE profile of everolimus, with the majority of these events being mild or moderate in severity [12]. Furthermore, the tolerability and AE profile of everolimus was maintained during the extension phase of this study [69].

**Fig. (3):**
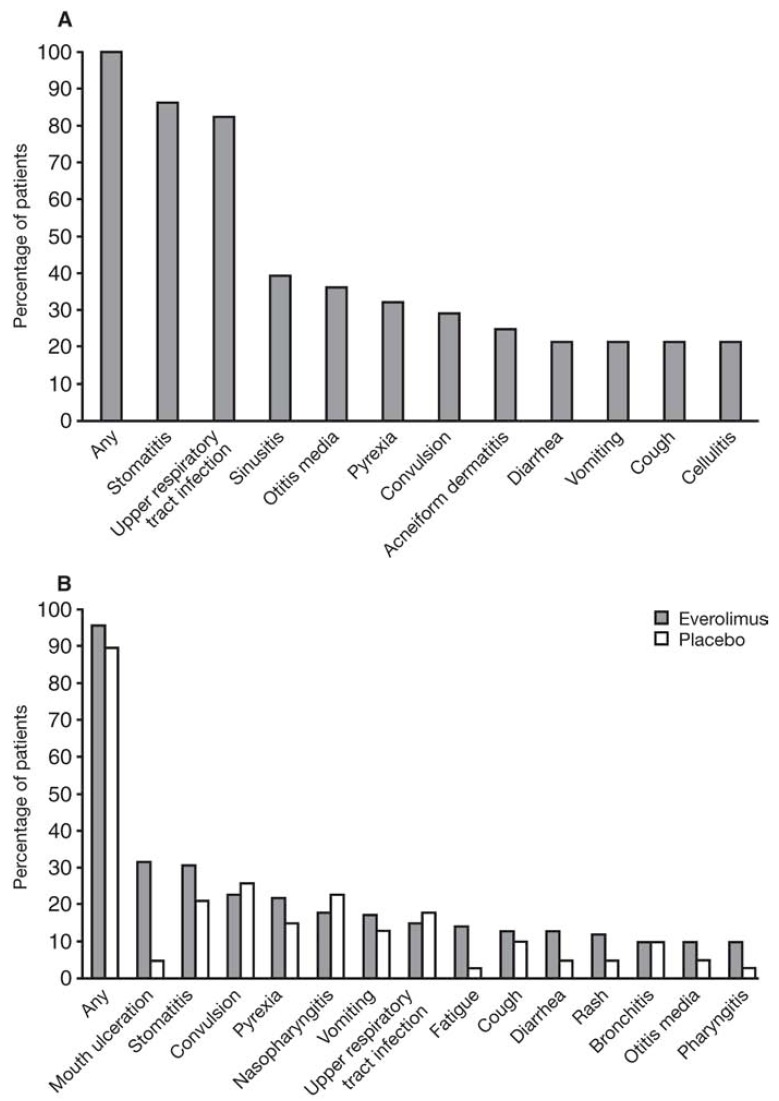
Treatment-emergent adverse events reported in (a) a phase II trial and (b) the phase III EXIST-1 trial of everolimus in patients with subependymal giant cell astrocytoma (SEGA) associated with tuberous sclerosis complex (TSC) [69]. “Reproduced from Curran MP.: Everolimus in patients with subependymal giant cell astrocytoma associated with tuberous sclerosis complex. Pediatr Drugs 2012: 14 (1): 51-60, with permission from Springer International AG (© 2012. All rights reserved).”

In the phase III EXIST-1 study, [69] the majority of AEs were grade 1 or 2 in severity, and the AE profile was consistent with that which was reported in the phase II C2485 trial (Fig. **3**) [12]. In EXIST-1, the most frequent grade 3 or 4 AEs that occurred in everolimus versus placebo recipients included stomatitis (8% vs. 3%), fever (6% vs. 0%), and convulsions (both 5%). No cases of AE-related treatment discontinuation were reported.

The phase III EXIST-2 trial assessed everolimus in patients with AML associated with TSC or sporadic LAM. The majority of AEs in this study were grade 1 or 2 in severity, and AEs were consistent with previously reported AE profiles of everolimus in patients with TSC. The most commonly reported AEs (≥20% of patients) in the everolimus treatment arm included stomatitis, nasopharyngitis, hemorrhage, cytopenia, acne, headache, cough, and hypercholesterolemia. Furthermore, the incidence of serious AEs was similar in the everolimus (19.0%) and placebo (17.9%) treatment groups.

## ROLE OF mTOR INHIBITORS IN THE MANAGEMENT OF TSC

Significant advances have been made over the past decade in understanding the molecular pathophysiology of TSC and, as a consequence, in identifying and developing targeted agents for this disorder. Although TSC affects multiple organ systems, a large proportion of TSC-related morbidity and mortality can be attributed to abnormalities in the brain, which may result in major neurological, cognitive, and behavioral manifestations [77]. 

An increased understanding of the role that the mTOR signaling pathway plays in TSC has been a major step in identifying the therapeutic potential of mTOR inhibitors such as sirolimus and everolimus. Phase II and III clinical trials have since been conducted and data from these studies indicate that mTOR inhibitors may be effective in the treatment of a variety of TSC manifestations, including SEGAs, LAM and AML [12, 55-57, 59, 69]. To date, everolimus is the only mTOR inhibitor to be approved for the treatment of patients aged ≥3 years with SEGA associated with TSC who require therapeutic intervention but are not candidates for curative surgical resection [13, 14]. This was based on clinical efficacy data from the pivotal phase II C2485 trial [12].

Despite the positive findings of these clinical trials, larger phase II and III studies are needed to clarify the role of m-TOR inhibitors in the management of TSC disease manifestations; a new trial evaluating the efficacy and safety of everolimus in focal epilepsy secondary to TSC is scheduled to begin in the near future [19]. Furthermore, it is unclear if prolonged use may lead to treatment resistance, and lack of long-term data means that the biological effect of continual treatment with mTOR inhibitors is unknown; while current evidence suggests that long-term mTOR inhibition may be required for maintenance of disease control, continual treatment may cause unwanted activation of other pathways [19, 71]. There is also insufficient evidence on the correlation between treatment response to mTOR inhibitors and specific clinical features or types of mutations [19]. Although mTOR inhibitors have the potential to treat the entire spectrum of manifestations associated with TSC, there are a number of outstanding clinical issues surrounding the use of these targeted agents, including when mTOR inhibitor therapy should be initiated and how to best utilize these agents to for the treatment of specific TSC-associated manifestations [77]. Moreover, the long-term efficacy, safety, and tolerability of mTOR inhibitors are not well defined. These issues emphasize that a better understanding of the role of the mTOR pathway in TSC is required, which will hopefully be provided by data from ongoing trials of mTOR inhibitors in TSC.

At present, the majority of clinical data on the efficacy and safety of mTOR inhibitors in TSC have been provided by studies of everolimus. Ongoing phase III studies (EXIST-1 and EXIST-2)[69] of everolimus will hopefully provide positive clinical data, particularly in TSC patients with SEGA, AML, and epilepsy. Indeed, preliminary results of these studies suggest that everolimus has the potential to prevent or delay the need for surgery, suppress or reverse disease progression, and improve TSC-related cognitive, behavioral, and developmental disorders 

**Fig. (4):**
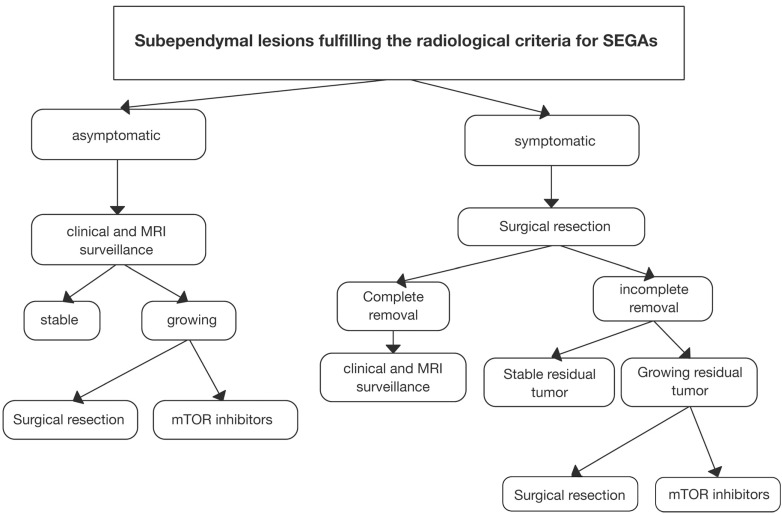
Suggested algorithm for the clinical management of subependymal giant cell astrocytoma (SEGA) associated with tuberous sclerosis complex. Reproduced with permission from Childs Nerv Syst 2011; 27(8): 1203-10 [74].

The early detection of TSC-related lesions such as SEGAs is a critical step that may allow the timely and effective implementation of surgical or medical intervention. An algorithm for the management of SEGAs in TSC has been proposed (Fig. **4**), [74] which takes into account the risks and benefits of current treatment options. Based on this algorithm, mTOR inhibitors may be recommended when asymptomatic SEGA is observed to be growing in two subsequent MRI evaluations, or as initial treatment to facilitate subsequent surgery in patients with bilateral lesions [74]. However, the immunosuppressant activity of everolimus means that it is necessary to cease treatment with the drug before surgery to avoid complications. Pharmacotherapy with agents such as mTOR inhibitors may be useful in situations where SEGAs present in an atypical location, exhibit aggressive growth, or in cases of re-growth where a second surgery is associated with a higher risk of complications. Furthermore, the concomitant presence of a growing AML and of intractable seizures should be taken into account when clinicians are making decisions between the two treatment options for an individual patient. Further studies are required to assess the long-term efficacy and safety of mTOR inhibitors and if prolonged use has the ability to prevent re-growth of TSC-related lesions. The optimal duration of treatment has also yet to be established, and strategies involving the use of longer treat durations at minimum dosages may warrant investigation. 

## CONCLUSIONS

With the rapidly increasing knowledge being gained on the mTOR pathway in relation to TSC, the future use of mTOR inhibitors in the treatment of TSC is likely to become well established. The positive effect that mTOR inhibitors have on a wide variety of TSC disease manifestations makes these drugs a potentially favorable treatment option. Moreover, starting the treatment at an early age, possibly even at infancy, might prevent the development of tumors, epilepsy, and other disease manifestations associated with TSC. In light of the promising clinical efficacy and safety that has been reported, it is anticipated that mTOR inhibitors such as sirolimus and everolimus can be an effective treatment option in TSC, and have the potential to be a disease-modifying therapy in patients with the disease. Further data from larger, prospective trials will help to establish the clinical efficacy, optimal dosage regimens, and safety profile of mTOR inhibitors in TSC, and more clearly define their role in this setting.

## Figures and Tables

**Fig. (1):**
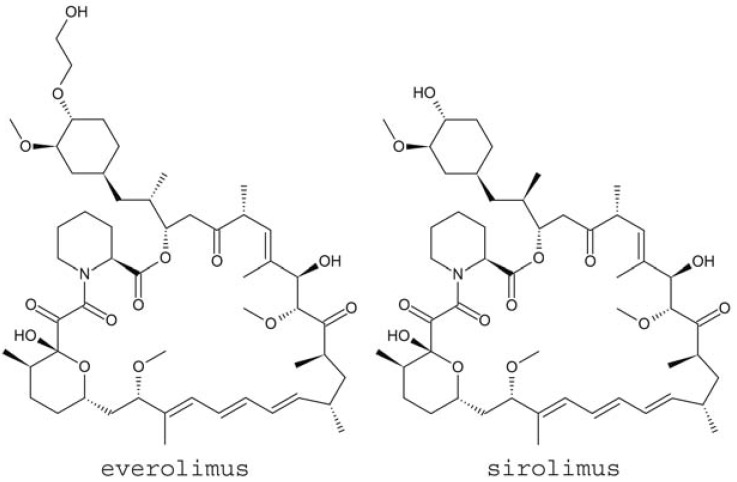
The chemical structures of the inhibitors of the mammalian target of rapamycin (mTOR) pathway, everolimus and sirolimus.

**Table 1.** Summary of the Key Pharmacokinetic Parameters of Oral Sirolimus and Everolimus. Reproduced from Expert Review of Anticancer Therapy, August 2011, Vol. 11, No. 8, Pages 1181-1192 with permission of Expert Reviews Ltd [19]

Characteristic	Sirolimus	Everolimus
Molecular weight (Kd)	914	958
Solubility in water (µg/mL)	2.6	8-fold higher
Hydrophobicity	Extreme	Less
Bioavailability (%)	1.6†	≥11†
Blood:plasma ratio (%)		26% free
Brain accumulation‡	5.7:1	3.1:1
t_max_ (h)	0.8	1-2
t_½_ (h)	86	30
V_ss_ (L/kg)	23	44-52
Clearance (mL/h•kg)	256 (man)	1200 (rat)

† Varies with formulation, dose and comedications.

‡ Ratio of drug concentration in brain (ng/g)/ drug concentration in blood (ng/mL) following 6 days of oral dosing at 3 mg/kg in rats.

**Table 2.** Ongoing Studies of mTOR Inhibitors in Tuberous Sclerosis Complex (TSC) [19]

Trial (ClinicalTrials.gov identifier)	Patient Population	Planned Enrolment	Study Design	Dosing Regimen	Primary Endpoint(s)
EXIST-1 (NCT00789828)	SEGA (all ages) associated with TSC	117	International, multicenter, randomized, double-blind, parallel-group, placebo-controlled, Phase III	Initial everolimus dose of 4.5 mg/m^2^/day titrated to achieve whole blood trough concentration of 5-15 ng/mL (dose adjustments based on safety and blood trough concentration) or placebo	SEGA response rate
EXIST-2 (NCT00790400)	Age ≥ 18 years with AML associated with TSC or sporadic LAM	99	International, multicenter, randomized, double-blind, placebo-controlled, Phase III	Everolimus 10 mg/day or placebo	AML response rate
SEGA (NCT00411619)	Age ≥ 3 years with TSC-associated SEGA	25	Open-label, Phase I/II	Everolimus 3 mg/m^2^/day titrated to achieve serum trough concentration of 10-15 ng/mL	Incidence of reported and observed adverse events
AML (NCT00457964)	Age 18-65 years with AML associated with TSC or sporadic LAM	30	Open-label, Phase I/II	Everolimus 5 or 10 mg/day, or 30, 50, or 70 mg/week	Proportion of patients with an AML response
AML long-term (NCT00792766)	Age 18-65 years with AML associated with TSC or sporadic LAM	10	Open-label, Phase I/II	Everolimus 5 or 10 mg/day, or 30, 50, or 70 mg/week	Everolimus tolerance
Epilepsy (NCT01070316)	Age ≥ 2 years with history of TSC-related epilepsy	20	Open-label, multicenter, Phase I/II	Everolimus 5 mg/m^2^/day titrated to achieve serum trough concentration of 5.1-10 ng/mL	Proportion of patients with a reduction in seizure frequency
Sirolimus in AML (NCT01217125)	Age ≥ 10 years with associated with AML	18	Open-label, Phase IV	Sirolimus titrated to achieve plasma levels of 4-8 ng/mL	AML volume
Neurocognition in TSC (NCT01289912)	Age 6-21 years with TSC	50	Multicenter, randomized, double-blind, parallel-group, placebo-controlled, Phase II	Everolimus 4.5 mg/m2/day or placebo	Neurocognition and Grade 3 and 4 adverse events

AML, angiomyolipoma; LAM, lymphangioleiomyomatosis; SEGA, subependymal giant-cell astrocytoma; TSC, tuberous sclerosis complex.
